# Fluoxetine, an antidepressant, suppresses glioblastoma by evoking AMPAR-mediated calcium-dependent apoptosis

**DOI:** 10.18632/oncotarget.3243

**Published:** 2014-12-31

**Authors:** Kao-Hui Liu, Shun-Tai Yang, Yen-Kuang Lin, Jia-Wei Lin, Yi-Hsuan Lee, Jia-Yi Wang, Chaur-Jong Hu, En-Yuan Lin, Shu-Mei Chen, Chee-Kin Then, Shing-Chuan Shen

**Affiliations:** ^1^ Taipei Medical University, College of Medicine, Graduate Institute of Medical Sciences, Taipei, Taiwan; ^2^ Taipei Medical University-Shuang Ho Hospital, Department of Neurosurgery, Taipei, Taiwan; ^3^ Taipei Medical University, Biostatistics Center, Taipei, Taiwan; ^4^ National Yang-Ming University, Department and Institute of Physiology, Taipei, Taiwan; ^5^ Taipei Medical University, College of Medicine, School of Medicine, Department of Physiology, Taipei, Taiwan; ^6^ Taipei Medical University-Shuang Ho Hospital, School of Medicine, Department of Neurology, Taipei, Taiwan; ^7^ Taipei Medical University Hospital, Department of Neurosurgery, Taipei, Taiwan; ^8^ Taipei Medical University-Wan Fang Hospital, Department of Neurosurgery, Taipei, Taiwan; ^9^ Taipei Medical University, College of Medicine, School of Medicine, Taipei, Taiwan

**Keywords:** glioblastoma, antidepressant, AMPA receptor, excitotoxicity

## Abstract

The efficacy of glioblastoma chemotherapy is not satisfactory; therefore, a new medication is expected to improve outcomes. As much evidence shows that antidepressants decrease cancer incidence and improve patients' quality of life, we therefore attempted to explore the potential for fluoxetine to be used to treat GBM and its possible underlying mechanism. The expression level of α-amino-3-hydroxy-5-methyl-4-isoxazolepropionic acid receptor (AMPAR) was determined using immunohistochemical staining and PCR analysis. The mechanism of fluoxetine-induced apoptosis of gliomas was elucidated. Computer modeling and a binding assay were conducted to investigate the interaction of fluoxetine with the AMPAR. The therapeutic effect of fluoxetine was evaluated using an animal model. We found that fluoxetine directly bound to AMPAR, thus inducing transmembrane Ca^2+^ influx. The rise in the intracellular calcium concentration ([Ca^2+^]_i_) causes mitochondrial Ca^2+^ overload, thereby triggering apoptosis. AMPARs are excessively expressed in glioma tissues, suggesting that fluoxetine specifically executes glioma cells. Our *in vivo* study revealed that fluoxetine suppressed the growth of glioblastomas in brains of Nu/Nu mice, an effect similar to that produced by temozolomide. Our preclinical studies suggest fluoxetine, a commonly used antidepressant, might be selectively toxic to gliomas and could provide a new approach for managing this disease.

## INTRODUCTION

The median survival time of grade IV glioma patients is approximately 12~15 months [1-4]. From both clinical and therapeutic aspects, the poor prognosis with gliomas involves different factors such as accessibility to surgery, radiotherapy, and chemotherapy [1, 4]. The anatomic location of gliomas makes it very difficult to surgically remove them, and it is impossible to avoid any damage to vital brain regions with radiotherapy. Moreover, the efficacy of chemotherapy is often impeded by the poor efficacy of drug delivery due to the diffusion barrier maintained by the blood-brain-barrier (BBB); it was reported that nearly 98% of small molecules and 100% of large molecules tested are incapable of passing through the BBB [4-6]. Unfortunately, the limited number of medications that are able to pass through the BBB cannot efficiently differentiate between healthy and cancerous cells [3, 6]. This causes serious side effects. Therefore, a drug which can pass through the BBB and selectively kill brain tumor cells is highly necessary.

Similar to other cancers, gliomas possess aberrant cell signaling that enhances their malignant behavior [7, 8]. Glutamate receptors, which are important for survival, differentiation, proliferation, and migration of cells during neural development, are highly expressed in gliomas, and are correlated with the malignancy of gliomas [9]. Recent reports showed that glutamate receptor antagonists inhibit cell proliferation of colon cancer, breast cancer, and lung cancer, and that the antiproliferative effect of the glutamate receptor antagonists is ascribed to their suppressive effect on cell division concomitant with an increase in cell death [9-11]. Nevertheless, due to their poor efficacy in penetrating the BBB, the glutamate receptor antagonists have had limited success in clinical applications to brain tumors [10].

Antidepressants are commonly prescribed for cancer patients suffering from depressive disorders that develop in later stages. Recently, retrospective studies showed that tricyclic antidepressants (TCAs) reduce the cancer risk of gliomas and that a selective serotonin reuptake inhibitor (SSRI), used as a antidepressant, has an antiproliferative or cytotoxic effect on certain cancers, due to its ability to pass through the BBB and directly carry out its pharmaceutical effects in regions of the brain. It should be noted that most antidepressants are much safer than chemotherapeutic agents. With these characteristics, antidepressants are promising leads for glioma treatment. Therefore, we attempted to explore the potential for antidepressants to be used to treat GBM and its possible underlying mechanism.

The intracellular Ca^2+^ concentration ([Ca^2+^]_i_) is well-controlled in the cytoplasm, is well-regulated across organelles under physiological conditions, and is involved in many biological functions such as secretion, contraction, metabolism, excitation, etc [12]. However, an abnormally prolonged increase in [Ca^2+^]_i_ may induce cell damage, and even trigger cell death [13]. Although the pathways underlying cell death are complicated, mechanisms associated with changes in the mitochondrial membrane permeability were shown to play important roles in cell death triggered by a mitochondrial matrix Ca^2+^ overload [14, 15]. Interestingly, it was shown that an SSRI increased [Ca^2+^]_i_ in different cancer cells such as oral cancer cells, prostate cancer cells, and bladder cancer cells, and the elevation of [Ca^2+^]_i_ was mediated by either transmembrane Ca^2+^ influx or Ca^2+^ release from the internal stores, or both [16-18]. Therefore, we hypothesized that fluoxetine, one of the most prescribed SSRI, will increase the [Ca^2+^]_i_, thereby triggering apoptosis in gliomas. In this study, we provide evidence showing a novel antitumor function of fluoxetine in gliomas and its possible underlying molecular mechanisms.

## RESULTS

### Fluoxetine-induced glioblastoma cell death is Ca^2+^-dependent and requires transmembrane Ca^2+^ influx

**Fig. 1:**
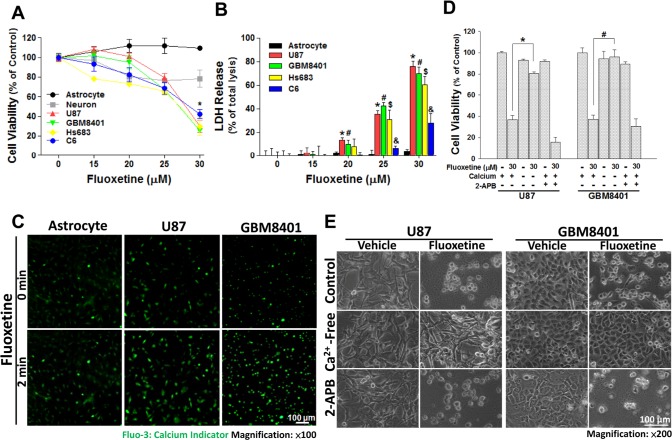
Fluoxetine-induced glioblastoma cell death is Ca-dependent and requires transmembrane Ca^2+^ influx; Ca^2+^ release from internal stores may have a minor contribution Cells were treated with the indicated concentrations of fluoxetine for 24 h, and the viability of cells was determined by an MTT assay (A) and LDH release assay (B). (C) Fluorescence imaging of [Ca^2+^]_i_ using Fluo-3 was conducted before and after 30 μM fluoxetine treatment. The image was taken in same area. A marked increase in the fluorescence intensity was seen in cells exposed to fluoxetine compared to the control. (D, E) Cell death induced by fluoxetine requires extracellular Ca^2+^. Glioblastoma cells were either cultured in normal medium or Ca^2+^-free medium, or pretreated with 2-APB, and then exposed to 30 μM fluoxetine for 24 h. Cell death was either quantified by an MTT assay (D) or observed by microscopy (E). Data were collected from three independent experiments, and are expressed as the mean ± SD. Results were statistically analyzed by Student's *t*-test; ^*^, ^#^, ^$^, ^&^ *p*<0.001 compared to the control group

Therapeutic doses of fluoxetine for depression patients are in the range of 20-60 mg/day and brain concentration of fluoxetine can reach up to about 30 μM [19-22]. Thus, we first investigated the effects of 0-30 μM fluoxetine application on the viability of glioma cells. Using glioma cell lines derived from both rats (C6) and humans (U87, GBM8401, and Hs683) as models, we found that 25-30 μM fluoxetine application decreased the viability of glioma cells, but had no effect on rat primary astrocytes and neurons. Both the MTT and LDH assays showed similar results (Fig. 1A-B).

Previous studies showed that SSRI antidepressants increase [Ca^2+^]_i_ in a variety of cells such as oral cells, prostatic cells, and prefrontal cortex astrocytes and that a sustained increase in [Ca^2+^]_i_ induces cell damage and may even cause cell death [16-18]. However, little is known about the role of Ca^2+^ signaling in fluoxetine-induced death of glioma cells. We therefore examined whether fluoxetine-induced glioma cell death was associated with changes in [Ca^2+^]_i_. Fluorescence Ca^2+^ imaging with Fluo-3-AM revealed that fluoxetine evoked an increase in [Ca^2+^]_i_ in U87 and GMB8401 cells, but not in rat primary astrocytes (Fig. 1C). To further determine what pathways (i.e., transmembrane Ca^2+^ influx vs. Ca^+2^ released from internal Ca^2+^ stores) contribute to fluoxetine-induced [Ca^2+^]_i_ elevation, cells were placed in medium with zero Ca^2+^ added or pretreated with 2-APB, a blocker of the ER calcium channel, followed by fluoxetine application. As shown in Fig. 1D-E, fluoxetine exhibited less cytotoxicity toward cells cultured in calcium-free medium, but retained a similar toxic effect following pretreatment with 2-APB. Collectively, these data suggest that the influx of extracellular Ca^2+^, not the internal release of Ca^2+^, plays an important role in fluoxetine-induced glioma cell death.

### Fluoxetine induced transmembrane calcium influx and subsequent glioblastoma cell death through its interaction with AMPARs

**Fig. 2:**
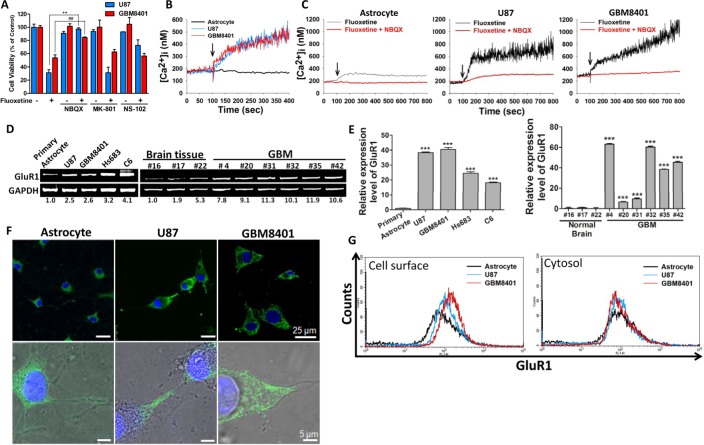
Fluoxetine induced transmembrane calcium influx and subsequent glioblastoma cell death through its interaction with AMPARs (A) Cells were pretreated with NBQX, NS-102 or MK-801 and then exposed to fluoxetine. Cell viability was quantified by an MTT assay. Only NBQX protected glioblastoma cells from fluoxetine-induced cell death. Data were collected from three independent experiments, and are expressed as the mean ± SD. Results were statistically analyzed by Student's *t*-test; ^**^, ^##^ *p*<0.01, compared to the control group. (B-C) Time course measurements of [Ca^2+^]_i_, using fluorescence spectrophotometry, made from Fura-2-loaded cells cultured in the absence or presence of 50 μM NBQX. Arrows indicate time of the addition fluoxetine (30 μM). Treatment with NBQX abrogated the [Ca^2+^]_i_ elevation induced by fluoxetine treatment. (D-E) mRNA expression levels of GluR1, an AMPAR subunit, were analyzed in cultured cells and in brain tissue samples from patients by RT-PCR (D), or a real-time PCR (E) The numbers below the bands indicate the relative intensities normalized to loading control. Results were statistically analyzed by Student's *t*-test; ^***^p<0.001 compared to the primary astrocyte or normal brain tissue #16, as a represented normal control. (F) In astrocyte, most of GluR1 proteins were expressed in the cytosol. In glioblastoma cell, most of GluR1 protein was expressed on the cell membrane. Blue: DAPI, Green: GluR1. (G) The cell surface (non-permeabilized) and cytosolic (permeabilized) GluR1 expression level on glioblastoma cell lines and astrocyte were analyzed by flow cytometry.

Multiple subtypes of glutamate receptor were shown to be expressed in glioblastoma specimens, and are critical in promoting the malignancy of gliomas [9]. Ionotropic glutamate receptors, in particular the AMPA receptor (AMPAR), NMDA receptor, and kinate receptor, are Ca^2+^-permeable. To determine which subtype(s) of glutamate receptor participated in fluoxetine-induced Ca^2+^ influx, we pretreated glioma cells with a specific antagonist (i.e., NBQX, an AMPAR blocker, MK-801, an NMDA receptor blocker, or NS-102, a kinate receptor blocker) followed by fluoxetine application, and then measured the cell viability. We found that pretreatment with NBQX, but not MK-801 or NS-102, increased the viability of glioma cells exposed to fluoxetine (Fig. 2A). Fluorescent Ca^2+^ imaging with Fura-2-AM revealed that fluoxetine elevated [Ca^2+^]_i_ in glioma cells within seconds, and the elevation of [Ca^2+^]_i_ was abolished when cells were pretreated with NBQX (Fig. 2B-C).

### GluR1, a subunit of AMPAR, is highly expressed in gliomas

**Fig. 3:**
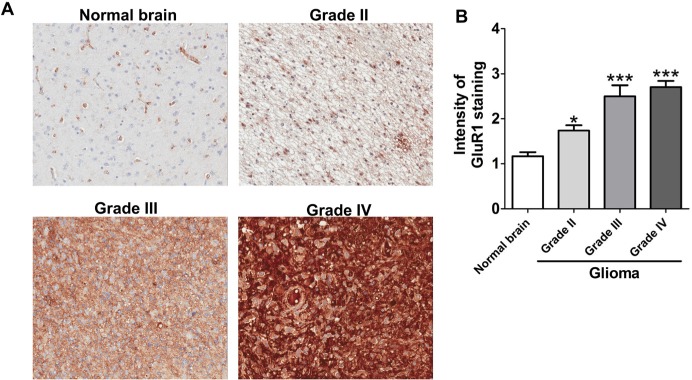
GluR1 upregulated in glioma patient tissues (A) Representative images of IHC analysis of normal brain and different grades of gliomas stained with anti-GluR1 antibody. (B) Comparison of GluR1 expression on different grades of human glioma and normal brain tissues by immunohistochemistry (IHC) staining. The human tissue microarray were obtained from US Biomax and contained normal brain (n=18) and grade II (n=73), grade III (n=20), grade IV (n=51) gliomas. ^*^p<0.05, ^***^p<0.001 when compared with the normal brain group.

The AMPAR is a tetramer assembled in various combinations from four subunits, GluR1~GluR4 [23, 24]. The GluR1 expression level is important for AMPAR-mediated calcium influx [25]. To determine the causal relationship between the GluR1 expression level and the malignancy of glioblastomas, both a RT-PCR and a real-time PCR analysis were used to measure the GluR1 expression level. We found that the expression level of GluR1 was elevated in both glioma cell lines and patient specimens, compared to controls (Fig. 2D-E). Moreover, the immunofluorescence image shown that GluR1 in glioblastoma cell were mainly expressed on the cell surface. On the contrary, in astrocyte, GluR1 were expressed mostly in the cytosol (Fig. 2F). The flow cytometry analysis also confirmed that GluR1 expression level in glioblastoma cell lines were higher than in astrocytes (Fig. 2G). The ratio of AMPAR on the cell membrane to intracellular pool is highly regulated. In fact, AMPAR could only transport calcium when it located on the cell membrane [26]. It indicated that functional (membrane) GluR1 expression level in glioblastoma cell line were higher than that in astrocyte. We next used immunohistochemical (IHC) staining to determine the GluR1 protein expression level by analysis tissue microarray from a commercial source (US Biomax) that contained normal brain (n=18) and different grades of gliomas (n=144). Immunohistochemical staining of the brain sections revealed that the GluR1 expression level is positively correlated with increased grades of gliomas (Fig. 3A-B). Taken together, these data suggested that fluoxetine-induced calcium influx and cell death of glioblastomas were mediated by AMPARs, which are highly expressed in glioblastomas.

### Fluoxetine induces mitochondrial membrane damage and activates the intrinsic apoptotic pathway through its interaction with GluR1

**Fig. 4:**
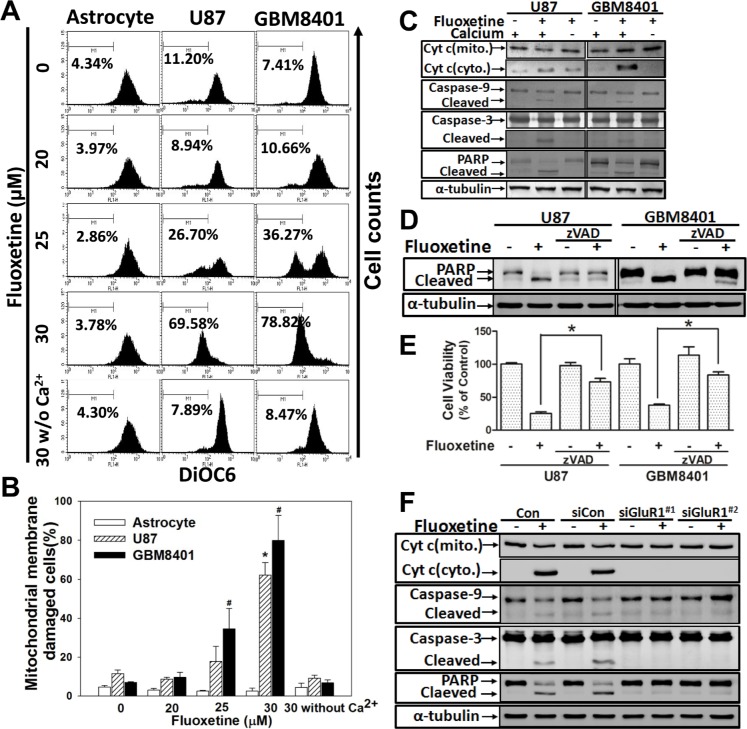
Fluoxetine induces mitochondrial membrane damage and activates the intrinsic apoptotic pathway through its interaction with GluR1 (A-B) Cells were treated with fluoxetine for 24 h, and DiOC6 staining was used to examine the damage done to mitochondrial membranes. The percentage of mitochondrial membranes damaged cells, which are within M1, is shown on each panel of the figure and summarized in histograms. Data were collected from three independent experiments, and are expressed as the mean ± SD. Results were statistically analyzed by Student's *t*-test. ^*, #^ *p*<0.001 compared to the control group. (C) A Western blot analysis of cytochrome c in both cytosolic and mitochondrial fractions, pro- and cleaved caspase-3 and -9, and PARP cleavage of glioblastoma cells treated with fluoxetine in the absence or presence of Ca^2+^ in the medium. (D-E) A Western blot analysis of PARP cleavage and a MTT assay to exam pan-caspase inhibitor, zVAD, effects on fluoxetine-induced apoptosis (F) A Western blot analysis of proteins as described in (C) was made from cells after transfection with the control siRNA or with GluR1 siRNA, followed by fluoxetine (30 μM) treatment.

Rapid [Ca^2+^]_i_ elevation induces mitochondrial calcium overloading and damages mitochondrial membranes, causing the release of apoptogenic factors, thereby triggering the apoptotic pathway [13-15]. To examine the effect of fluoxetine-induced Ca^2+^ influx on mitochondrial integrity, we examined the MMP and subcellular distribution of cytochrome c. Our flow cytometric analysis showed that fluoxetine respectively decreased the MMP by 62.11%±6.47% and 79.83%±12.86% in U87 and GBM8401 cells (Fig. 4A-B). The Western blot analysis revealed that after fluoxetine treatment, a significant amount of cytochrome c was released from the mitochondrial matrix and was present in the cytosolic fraction, accompanied by activation of caspase-9, caspase-3, and poly (ADP-ribose) polymerase (PARP). On the other hand, removal of Ca^2+^ from the extracellular medium during fluoxetine application abolished the apoptotic events as described above (Fig. 4C). The pan-caspase inhibitor, zVAD, blocked fluoxetine-induced PARP cleavage and reversed the fluoxetine-induced cell viability decrease corroborating the suggestion that fluoxetine induces apoptosis in a caspase-dependent manner (Fig. 4D-E).

To further confirm the notion that AMPARs play an important role in fluoxetine-induced apoptosis, the same experiments as shown in Fig. 4C were conducted in glioma cells in which GluR1 was knocked down by siRNA. Fluoxetine-induced apoptosis was blocked by GluR1 knockdown (Fig. 4F). These data suggest that apoptosis triggered by fluoxetine involves a mitochondrion-dependent pathway and contains GluR1 through its interaction with AMPARs.

### Fluoxetine directly binds to the GluR1

**Fig. 5:**
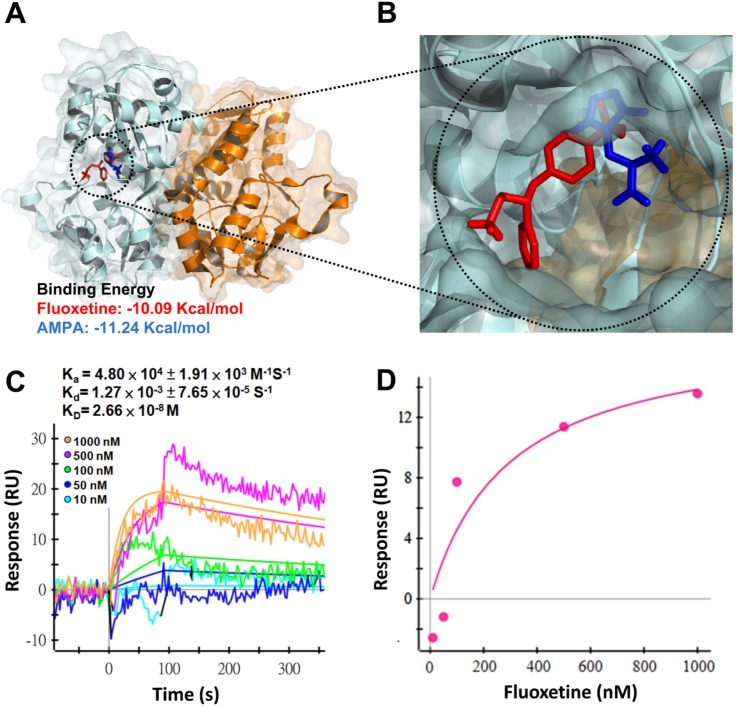
Fluoxetine directly binds to the GluR1 (A and B) Computer modeling of protein-ligand interaction among GluR1 ligand-binding domain (LBD) and ligands. Localization of the protein-ligand binding site and estimation of free energy required for the binding were determined using AutoDock. The predicted structure and estimated free energy of GluR1 LBD docked with either fluoxetine or AMPA were modeled. (C) Surface plasmon resonance (SPR) sensorgram shows the association and dissociation between fluoxetine and GluR1. Indicated concentrations of fluoxetine were injected into the sensor chip. Binding is expressed as the differential response unit (RU) between the bindings of fluoxetine to the GluR1-immobilized sensor chip or to a blank sensor chip. Results show that the apparent association rate constant (K_a_) was 4.8 × 10^4^ M^−1^ s^−1^ and the dissociation rate constant (K_d_) was 1.27× 10^−3^ s^−1^, giving an equilibrium dissociation constant (K_D_) of 2.66 × 10^−8^ M. (D) The equilibrium-state response unit was plotted versus the concentration of fluoxetine.

To determine whether fluoxetine can act as a ligand and directly bind to GluR1, the interaction of fluoxetine with GluR1 was simulated by computer modeling. An *in silico* study showed that both fluoxetine and AMPA had similar properties of free binding energies and shared the same binding sites, i.e., they both bound to the same ligand-binding domain of GluR1 (Fig. 5A-B). To further confirm the prediction of the computer simulation, we carried out surface plasmon resonance (SPR) spectroscopy to analyze the binding affinity and kinetic rate constants of ligand-receptor interactions. As shown in Fig. 5C-D, the sensorgram demonstrates that fluoxetine bound to GluR1 in a dose-dependent manner, with rate constants, K_a_ = 4.8 × 10^4^ M^−1^ s^−1^ and K_d_ = 1.27× 10^−3^ s^−1^, and with binding affinity, K_D_ = 2.66 × 10^−8^ M. These data indicate that fluoxetine can directly bind to GluR1.

### Fluoxetine suppressed the growth of glioblastoma cells *in vivo*

**Fig. 6:**
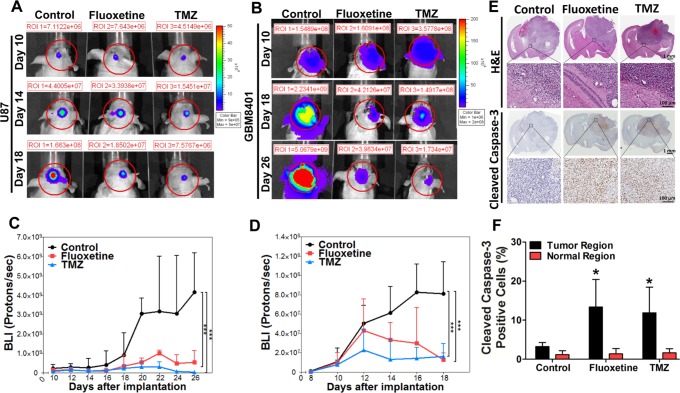
Fluoxetine suppressed the growth of glioblastoma cells *in vivo* without damaging normal brain regions Effects of fluoxetine or temozolomide (TMZ) on intracranial luciferase-expressing U87 (A and C) and GBM8401 (B and D) xenografts. Representative bioluminescence images obtained from experiments were shown. The region of interest (ROI) is marked by red circles. The results were statistically analyzed by two-way Repeated Measured ANOVA. The differences among control, Fluoxetine, and TMZ on tumor size at certain days were evaluated using Bonferroni post hoc analysis. ^***^p<0.001 when compared with the control group. (E) Representative images show glioblastoma formation after U87 cell implantation. Intracranial glioblastoma xenograft sections were examined by H&E staining and IHC staining. Micrographs showing immunostaining of cleaved caspase-3 in intracranial glioblastoma xenografts. (F) The percentage of cleaved caspase-3 positive cells was increased in the tumor region of Fluoxetine- or TMZ-treated groups. Results were statistically analyzed by Student's *t*-test. ^*^p<0.05 compared to the control group.

In order to explore the *in vivo* relevance of our findings obtained *in vitro*, we generated tumor xenografts by subcutaneously injecting 5 × 10^6^ U87 cells into nude mice. When the tumor size reached 100 mm^3^, the mice were administered fluoxetine (10 mg/kg/day, o.p.) or TMZ (5 mg/kg/day, i.p.), a GBM first-line clinical chemotherapeutic medicine, for comparison. After administration, the tumor was considerably reduced on day 6, and had become undetectable by day 12, compared to the controls. Both fluoxetine and TMZ treatments showed similar results (Supplementary Fig. S1). To address whether fluoxetine was able to pass through the BBB and carry out its antitumor activity intracranially, we implanted 10^6^ luciferase-expressing U87 cells or 2 × 10^5^ luciferase-expressing GBM8401 cells into the right striatum of nude mice. The bioluminescent signal revealed that intracranial tumor growth was significantly inhibited in fluoxetine- and TMZ-treated mice (Fig. 6A-D). The data showed that fluoxetine suppressed the growth of intracranial glioblastoma brain tumors.

To examine whether fluoxetine had damaging effects on normal brain tissues, mice brains from fluoxetine- and TMZ-treated mice and control mice were coronally sliced and then inspected after H&E staining or immunostaining (Fig. 6E). There was a large amount of cleaved caspase-3 in brain slices from both fluoxetine- and TMZ-treated mice compared to those of the control group (Fig. 6F). Most importantly, the cleaved caspase-3 signals were specifically expressed in the brain tumor region, while only weak signals were present in the normal brain region. These data suggests that fluoxetine specifically damages glioblastoma cells without having deleterious effects on normal brain regions.

## DISCUSSION

Gliomas are one of the most aggressive and common primary malignant brain tumors. The median survival time of a glioma patient who receives treatment is only about 12~15 months, indicating that current medication is not effective [1, 4]. One possible reason for this short survival time could be that many chemotherapeutic drugs commonly used to treat other cancers have poor efficacy in passing through the diffusion barrier of the BBB [4, 5]. This results in a marked reduction of drug delivery to the targeted brain region, thereby limiting its clinical application in brain tumor treatment [4]. On the other hand, the few drugs that are able to pass through the BBB cannot efficiently differentiate between healthy and cancerous cells, thus causing serious side effects. One typical example is TMZ, a first-line brain tumor chemotherapeutic drug. A drug which can pass through the BBB and specifically induce cell death in glioma cells has been highly anticipated.

**Fig. 7:**
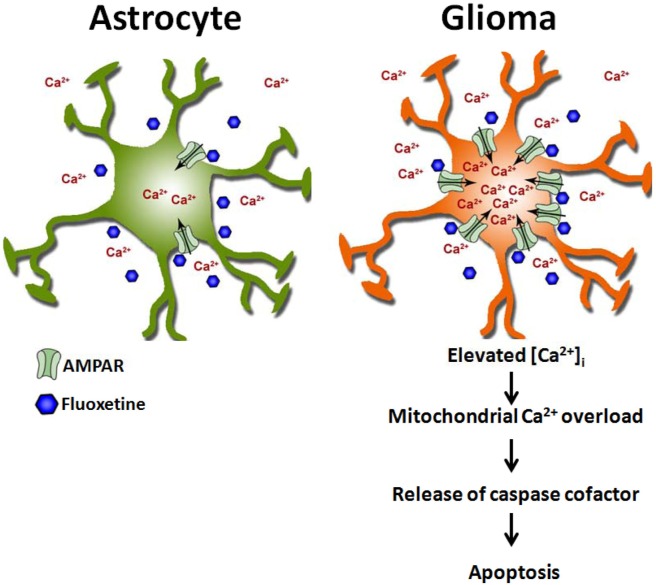
Schematic overview Fluoxetine activates AMPA receptors (AMPARs), which are highly expressed by glioma cells compared to neuroglia cells, causing transmembrane Ca^2+^ influx. The sustained increase in [Ca^2+^]_i_ induces mitochondrial membrane permeability change, resulting in the release of apoptogenic factors (e.g., cytochrome c) and the formation of apoptosomes, consequently leading to caspase activation.

This is the first study to show that AMPARs were excessively expressed in glioma cells and that fluoxetine, through its direct binding to GluR1, activated AMPARs and evoked robust Ca^2+^ influx. An acute increase in [Ca^2+^]i causes mitochondrial Ca^2+^ overload, thereby triggering apoptosis (Fig. 7). Moreover, our *in vivo* study showed that fluoxetine suppressed the growth of gliomas in brains of Nu/Nu mice, an effect similar to that produced by TMZ. Taken together, our study indicates that fluoxetine is a safe and potential drug that could provide a new approach for managing gliomas.

Fluoxetine, a specific SSRI, is one of the most popular antidepressants and has been used for decades. The mechanism by which fluoxetine treats depression has long been ascribed to its inhibitory action on serotonin transporters. Blockade of serotonin reuptake results in an increase in the serotonin concentration at synaptic clefts, which in turn activates neurons and relieves depressive symptoms. Interestingly, it was first shown by Abdul *et al*. using prostate carcinoma cell lines as a model that SSRIs, including both fluoxetine and zimelidine, inhibited the growth of human prostate cancer[27]. A study by Levkovitz *et al*. indicated that SSRI and MAOI antidepressants activate caspase-3 and induce apoptosis in rat glioma cell lines [28]. However, the underlying mechanisms were not elucidated. In 2010, Sivan Tzadok *et al*. showed a synergistic anti-proliferative effect of imatinib when co-applied with fluoxetine, sertraline, or perphenazine, as tested in human glioblastoma cell lines [29]. Since FDA-approved medications is more safety and cost-effective than new agents, the novel use of old drugs for cancer therapy became a more acceptable strategy in the pharmaceutical study [30, 31]. Recently, a new conceptually glioma treatment approach based on combining temozolomide with nine repurposed drugs, including a SSRI antidepressant, was proposed [32, 33].

Epidemiological studies conducted in both Britain and Canada showed that antidepressants reduce the cancer risk in colon cancer and glioma patients, although some studies revealed that antidepressants might increase the incidence of seizures [34-36]. Previous clinical trials indicated that paroxetine and citalopram markedly improved cancer patients' quality of life [37, 38]. Additionally, a retrospective review suggested that using SSRI antidepressants, including citalopram, escitalopram, fluoxetine, fluvoxamine, paroxetine, and sertraline, for glioma treatment did not adversely affect survival rates [39]. Based on those studies, we attempted to identify which SSRI antidepressants could be potentially used for treating glioma cells and studied the underlying mechanisms.

Ishiuchi *et al*. indicated that the expression level of Ca^2+^-permeable AMPARs was positively correlated with the malignancy of gliomas [40]. Their study found that malignant glioma cells secreted glutamate into the extracellular space in an autocrine or paracrine manner, resulting in the activation of Ca^2+^-permeable AMPAR and Akt pathways. This facilitated the growth of glioma cells and increased their ability to migrate. Our study showed that fluoxetine directly binds to AMPARs, causing an elevation in [Ca^2+^]_i_. The rise in [Ca^2+^]_i_ was so rapid that the mitochondrial matrix had already been overloaded with Ca^2+^ before activation of the Akt pathway occurred, and then as a consequence, apoptosis was triggered. Because AMPARs are excessively expressed by glioma cells, fluoxetine was expected to induce massive death of glioma cells through its interaction with AMPARs.

The monoamine hypothesis states that depression is due to the dysfunctional homeostasis of monoamine levels in the brain. It was previously believed that the administration of SSRI, MAOI, or TCA antidepressants increases the concentration of monoamines at the synaptic cleft and thus relieves depressive symptoms. Later, Svenningsson *et al*. proposed a different mechanism suggesting that fluoxetine might have a modulatory role on the dopamine- and cAMP-regulated phosphoprotein of M_r_ 32,000 (DAPRR-32) [41] and that an increase in phosphorylation of GluR1 at Ser-831 and Ser-845, through activation of these pathways results in facilitation of calcium influx, and activates the calcium/calmodulin-dependent kinase II pathway, which in turn enhances the expression of brain-derived neurotrophic factor, leading to a reduction in depressive symptoms.

We were the first group to report that fluoxetine directly binds to GluR1 to evoke Ca^2+^ influx, subsequently triggering apoptosis in glioma cells. Our findings differ from those of previous studies which showed that fluoxetine induces apoptosis in glioma cells in an indirect manner through pathways involving the phosphorylation of GluR1. Our findings, however, do not exclude those mechanisms; indeed, both mechanisms may coexist and have certain contributions to apoptotic signaling in glioma cells.

Substantial evidence indicates that Ca^2+^-permeable AMPARs play an important role in the etiology of many nervous system diseases such as stroke, epilepsy, and amyotrophic lateral sclerosis [42]. Although the molecular mechanism responsible for degeneration remains unclear, it is believed that neurons may secrete an enormous amount of glutamate into the extracellular space, which, once bound to Ca^2+^-permeable AMPARs, may induce massive cell death. Note that, if the mechanism of action of fluoxetine is similar to that of glutamate, then it is expected that fluoxetine will cause some damage to the nervous system; however, it has never been reported that the use of fluoxetine exacerbates nervous system diseases. This issue requires further investigation.

The most common sources of brain metastasis are lung cancer, breast cancer, colon cancer, renal cancer, and melanomas, among others. The survival time of patients is markedly reduced once brain metastasis occurs [43]. As previous epidemiological studies suggest that antidepressants might reduce cancer risks [34, 35, 37, 38], future investigations into the expression level of GluR1 in other cancer cells, especially for those in brain metastasis cancer, and the effect of fluoxetine on other cancers would be valuable. Given the data shown in this study indicating that fluoxetine is a relatively safe and potential pharmaceutical drug, further investigation is warranted.

## MATERIALS AND METHODS

### Glioma tumor samples

Tissue samples were collected at Wan Fang Hospital (Taiwan). All tissue samples and clinical information were obtained as part of an Institutional Review Board (IRB)-approved study of the molecular analysis on brain tumors at Taipei Medical University. Brain tissue samples included glioblastomas (*n* = 6) and those of the normal brain (*n* = 3).

### Cell culture

Glioblastoma U87, C6, and Hs683 cells were obtained from American Type Culture Collection and GBM8401 was obtained from Bioresource Collection and Research Center of Taiwan. Those cell lines were grown in Dulbecco's modified Eagle's medium (Invitrogen) supplemented with 10% heat-inactivated fetal bovine serum (Biological Industries). Primary cortical neuron and astrocyte cultures were prepared as previously described [44]. The human glioma cell lines were authenticated through cell morphology monitoring, growth curve analysis and short tandem repeat profiling analysis in 2014. The characterization of primary cortical neuron, primary astrocyte and C6 glioma cell line were confirmed by NeuN, GFAP or S100 expression, respectively. For the intracranial xenograft experiment, luciferase-expressing U87 and GBM8401 cells were established by transfection of a pGL4.51[*luc2*/CMV/Neo] vector (Promega) into the cell lines.

### Cell survival analysis

Cell viability was assessed by an MTT assay, and cytotoxicity was analyzed by a lactate dehydrogenase (LDH) assay. Briefly, cells were incubated with 0.25 mg/mL MTT (Sigma) at 37°C for 1 hr and the MTT-fomazan were measured spectrophotometrically (μQuant, Bio-Tek) at 595 nm after dissolution of the crystals in DMSO. The amount of LDH released was detected with a cytotoxicity detection kit (Roche). The culture medium was centrifuged, and the absorbance was detected at 530 nm using an ELISA reader. The percentage of cytotoxicity was determined by the equation: [(Experimental group - Control group) / (Triton-100-treated group - Control group)] ×100%.

### Intracellular calcium ([Ca^2+^]_i_) measurement

To measure the intracellular concentration, cells were suspended and incubated with 5 μM Fura-2-AM (Molecular Probes) for 45 min at 37°C. Cells were then placed in the cell chamber of a spectrofluorometer (Hitachi FL Spectrophotometer F-4500) equipped with dual excitation wavelengths of 340 and 380 nm to respectively measure the Ca^2+^-bound and -free forms of Fura-2 at an emission wavelength of 510 nm. The ratio of the fluorescence at the two wavelengths (A_340 nm_/A_380 nm_) was used to calculate changes in [Ca^2+^]_i_ as previously described [45].

To capture real-time [Ca^2+^]_i_ images, cells were loaded with 5 μM Fluo-3-AM (Molecular Probes) for 45 min, and then immediately examined on a fluorescence microscope (Olympus IX81 microscope).

### Measurement of the mitochondrial membrane potential

Cells were treated with the indicated concentration of fluoxetine for 24 h and then incubated with 40 nM DiOC6(3) (Sigma) for 30 min at 37°C. After treatment, cells were washed and suspended in PBS. DiOC6(3) fluorescence intensities were measured with a flow cytometer (FACScan, Becton Dickinson)

### Reverse-transcription polymerase chain reaction (RT-PCR) and a real-time PCR

Total RNA was extracted with the TRI reagent (Sigma) following the manufacturer's instructions. Complementary (c)DNA was subjected to PCR with primers that amplified GluR1-4 (GluR1-4 sense, CCTTTGGCCTATGAGATCTGGATGTG and common antisense, TCGTACCACCATTTGTTTTTCA). A second PCR was performed with primers specific for GluR1 (GluR1 sense, AAGAGGGACGAGACCAGACAAC and the common antisense one as used for first PCR) [40].

Real-time PCRs were carried out with specific primers (GluR1 sense: GACGCCGGACCAACTACAC and antisense: GCTGCAGGGACAAACTTATCA; and GAPDH sense: GAAATCCCATCACCATCTTCCAGG and antisense: GAGCCCCAGCCTTCTCCATG). The expression of GAPDH was used as an internal control.

### Immunofluorescence

For immunofluorescence analysis, cultured cells were properly fixed and permeabilized. After blocked by 1% BSA, cells were incubated with anti-GluR1 antibody overnight followed by incubated with FITC-conjugated anti-rabbit antibody (Santa Cruz Biotechnology). Nuclei were counterstained with DAPI. The images were acquired using laser confocal microscope (Leica, TCS SP5 Confocal Spectral Microscope Image System). For flow cytometry analysis, cultured cells were incubated with anti-GluR1 antibody under non-permeabilizing and permeablizing conditions followed by incubated with FITC-conjugated anti-rabbit antibody (Santa Cruz Biotechnology). Cells were washed and suspended in PBS. Fluorescence intensities were measured with a flow cytometer (FACScan, Becton Dickinson).

### Surface plasmon resonance (SPR) spectroscopy

SPR spectroscopy was used to analysis the binding affinity and kinetic rate constants of a set of small molecule interactions [46, 47]. Real-time biomolecular interactions were analyzed by a Bio-Rad ProteOn™ XPR 36 protein interaction array system (Bio-Rad). All procedures followed the manufacturer's instructions. Briefly, to evaluate the association and dissociation kinetics of fluoxetine and GluR1, increasing concentrations of fluoxetine (10, 50, 100, 500, and 100 nM) were diluted in PBST buffer and injected for 90 s followed by washing in PBST for 270 s. Sensorgrams for binding interactions were recorded in real time and analyzed after subtracting the blank channel. The association rate constant (K_a_), dissociation rate constant (K_d_), and equilibrium constant (K_D_) were calculated by ProteOn Manager software (Bio-Rad).

### Computational molecular docking

The protein structure of GluR1 in the docking study was predicted by homology modeling with Swiss-Model Workshop [48, 49]. AutoDock 4.2 was used to calculate the docking position and binding energy of fluoxetine or α-amino-3-hydroxy-5-methyl-4-isoxazolepropionic acid (AMPA) to GluR1 [50]. We carried out the Lamarckian genetic algorithm and set 2.5 × 10^6^ evaluations and 100 runs for docking. The lowest binding energy and docking position of fluoxetine and AMPA are visualized by The PyMol Molecular Graphics System (Schrödinger).

### Western blot analysis

Cell lysates were prepared by suspending cells in RIPA buffer (50 mM Tris-HCl at pH 7.4, 1% Nonidet P-40, 150 mM NaCl, 1 mM EGTA, 0.025% sodium deoxycholate, 1 mM NaF, 1 mM Na_3_VO_4_, and 1 mM PMSF). Equal amounts of protein were electrophoresed on 10% sodium dodecylsulfate-polyacrylamide gels, and transferred to polyvinylidene difluoride membranes (Millipore). Membranes were probed with specific antibodies (anti-cytochrome C, anti-α-tubulin (Neomarker), anti-caspase-9 (Cell Signaling), anti-caspase-3, anti-PARP (Imgenex), anti-GluR1 (Abcam) and then quantified by the colorimetric substrates.

### Tissue array

Human tissue array of normal and glioma brain tissues were obtained from US Biomax (Rockville, MD, USA). Tissues sections were stained with anti-GluR1 antibody (Abcam) and revealed using a polymer detection system kit (Leica Biosystems). The staining intensity was blind rated from 1 (weak staining) to 4 (strong intensity of staining) by pathologist.

### Animals

Male athymic nude mice at 5~6 weeks old were obtained from BioLasco (Taiwan) and maintained in a specific-pathogen-free facility. All experimental procedures were approved by the Institute of Animal Care and Use Committee of Taipei Medical University.

### Tumor xenografts

Each animal was anesthetized with an i.p. injection of xylazine (Sigma) and zolazepam (Zoletil 50, Virbac), and then secured in a stereotaxic frame. Luciferase-expressing U87 cells (10^6^ cells in PBS) or GBM8401 cells (2 × 10^5^ cells in PBS) were intracranially implanted into the right striatum. One week after implantation, mice were treated with fluoxetine (10 mg/kg/day, o.p., Sigma) or TMZ (5 mg/kg/day, i.p., Sigma). Luciferase activity was analyzed on the indicated day by IVIS-200 and Living Image Software (Caliper LifeSciences).

### Histology

Mice were subjected to deep anesthesia and then transcardially perfused with ice-cold PBS and 10% formaldehyde. Sections were stained with hematoxylin and eosin and rabbit-anti-cleaved caspase-3 (Abcam) and revealed using a polymer detection system kit (Leica Biosystems).

### Statistical analysis

Values presented in the study were repeated at least three times from three independent experiments and are expressed as the mean ± standard deviation. The significance of the difference from the respective controls for each experimental was assayed using Student's *t*-test or an analysis of variance for multiple-group experiments. *p* values of <0.05, <0.01, and <0.001 were considered statistically significant.

## SUPPLEMENTARY MATERIALS AND METHODS AND FIGURES


