# Supplementary material for: Fluoxetine, an antidepressant, suppresses glioblastoma by evoking AMPAR-mediated calcium-dependent apoptosis
Source: Oncotarget. 2014 Dec 31;6(7):5088–101. doi: 10.18632/oncotarget.3243 (PMC4467135; doi:10.18632/oncotarget.3243)
Supplement: Supplementary file 1 [file oncotarget-06-5088-s001.pdf]

Fluoxetine, an antidepressant, suppresses glioblastoma by evoking AMPAR-mediated calcium-dependent apoptosis

Supplementary Materials and Methods

Tumor xenografts

For the subcutaneous tumor model, animals were inoculated by a subcutaneous (s.c.) injection with U87 cells (5×10^6 cells in PBS). Animals were randomly assigned to various groups and treated with fluoxetine (10 mg/kg/day, o.p.) or temozolomide (TMZ) (5 mg/kg/day, intraperitoneally (i.p.)) when the tumor had reached an average size of 100 mm^3. Tumor sizes were measured with external calipers, and the volume was calculated as the (length/2) × $(width)^2$.

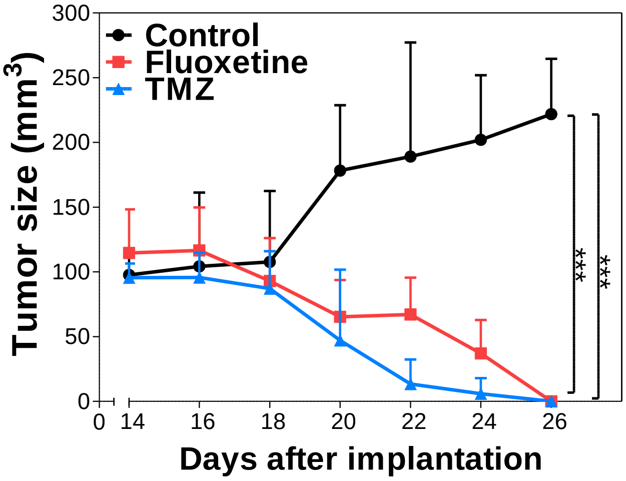

Supplementary Fig. 1: Fluoxetine suppressed the growth of glioblastoma cells *in vivo*. The effect of fluoxetine or temozolomide (TMZ) on tumor growth *in vivo*. The results were statistically analyzed by two-way Repeated Measured ANOVA. The differences among control, Fluoxetine, and TMZ on tumor size at certain days were evaluated using Bonferroni post hoc analysis. $^{***}p<0.001$ when compared with the control group.

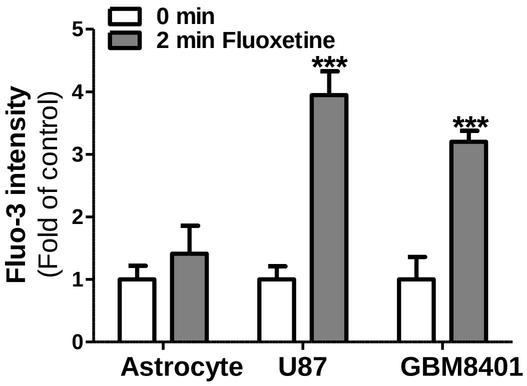

Supplementary Fig. 2: Fluoxetine specifically elevated the intracellular calcium concentration in AMPAR-expressing glioblastoma cell lines. Fluorescence imaging of $[Ca^{2+}]_i$ using Fluo-3 was conducted before and after 30 µM fluoxetine treatment. Summary histograms of Fluo-3 intensity were shown. A marked increase in the fluorescence intensity was seen in cells exposed to fluoxetine compared to the control (treatment at 0 min). The results were statistically analyzed by Student's *t*-test. $^{***}p<0.001$ when compared with the control (treatment at 0 min).
